# SCFβ‐TrCP ubiquitinates CHK1 in an AMPK‐dependent manner in response to glucose deprivation

**DOI:** 10.1002/1878-0261.12403

**Published:** 2018-12-03

**Authors:** Ying Ma, Danrui Cui, Xiufang Xiong, Hiroyuki Inuzuka, Wenyi Wei, Yi Sun, Brian J. North, Yongchao Zhao

**Affiliations:** ^1^ Key Laboratory of Combined Multi‐Organ Transplantation Ministry of Public Health First Affiliated Hospital Zhejiang University School of Medicine Hangzhou China; ^2^ Institute of Translational Medicine Zhejiang University School of Medicine Hangzhou China; ^3^ Department of Pathology Beth Israel Deaconess Medical Center Harvard Medical School Boston MA USA; ^4^ Center for Advanced Stem Cell and Regenerative Research Tohoku University Graduate School of Dentistry Sendai Japan; ^5^ Division of Radiation and Cancer Biology Department of Radiation Oncology University of Michigan Ann Arbor MI USA

**Keywords:** AMPK, CHK1, glucose deprivation, ubiquitination, β‐TrCP

## Abstract

The ATR/CHK1 pathway is a key effector of cellular response to DNA damage and therefore is a critical regulator of genomic stability. While the ATR/CHK1 pathway is often inactivated by mutations, CHK1 itself is rarely mutated in human cancers. Thus, cellular levels of CHK1 likely play a key role in the maintenance of genomic stability and preventing tumorigenesis. Glucose deprivation is observed in many solid tumors due to high glycolytic rates of cancer cells and insufficient vascularization, yet cancer cells have devised mechanisms to survive in conditions of low glucose. Although CHK1 degradation through the ubiquitin–proteasome pathway following glucose deprivation has been previously reported, the detailed molecular mechanisms remain elusive. Here, we show that CHK1 is ubiquitinated and degraded upon glucose deprivation by the Skp1‐Cullin‐F‐box (β‐TrCP) E3 ubiquitin ligase. Specifically, CHK1 contains a β‐TrCP recognizable degron domain, which is phosphorylated by AMPK in response to glucose deprivation, allowing for β‐TrCP to recognize CHK1 for subsequent ubiquitination and degradation. Our results provide a novel mechanism by which glucose metabolism regulates a DNA damage effector, and imply that glucose deprivation, which is often found in solid tumor microenvironments, may enhance mutagenesis, clonal expansion, and tumor progression by triggering CHK1 degradation.

**Abbreviations**
CDT2: DDB1‐ and CUL4‐associated factor 2
CHK1: checkpoint kinase 1
CHK2: checkpoint kinase 2
CHX: cycloheximide
CRL: Cullin‐RING E3 ubiquitin ligases
FBX6: F‐box protein 6
GD: glucose deprivation
NG: normal glucose
UCN‐01: 7‐hydroxystaurosporine
β‐TrCP: β‐transducin repeat‐containing protein

## 1 Introduction

Cancer cells have a high rate of glycolysis that they rely on for the generation of ATP even in the presence of sufficient oxygen. This is a characteristic of cancer cells known as the Warburg effect (Annibaldi and Widmann, 2010; Hanahan and Weinberg, 2011; Warburg, 1956). Glucose levels in solid tumors are relatively low and poorly distributed due to high glycolytic rate of cancer cells and poor distribution of glucose as a result of insufficient and disorganized vascularization (Bergers and Benjamin, 2003; Skinner *et al*., 1990). Although dependence on glucose for growth makes cancer cells vulnerable to glucose deprivation, they are still able to survive in conditions of low glucose. Previous studies have shown that due to the Warburg effect of cancer metabolism, solid tumors are often in a lactic acidosis microenvironment, which prolongs cancer cell survival under conditions of glucose deprivation (Denko, 2008; Wu *et al*., 2012). Furthermore, lactic acidosis alters cancer cell metabolism (Chen *et al*., 2008), induces chromosomal instability (Dai *et al*., 2013), and promotes tumor angiogenesis (Vegran *et al*., 2011). However, mechanistically, how cancer cells survive low‐glucose conditions and how these conditions promote tumorigenesis and metastatic transformation remains largely elusive.

Checkpoint kinase 1 (CHK1) is a Ser/Thr protein kinase that is involved in initiating checkpoints during the cell cycle in response to DNA damage. CHK1 is a key downstream regulator of the ataxia‐telangiectasia‐mutated‐and‐Rad3‐related kinase (ATR) response pathway to DNA damage and is phosphorylated directly by ATR leading to its activation (Liu *et al*., 2000). Similarly, checkpoint kinase 2 (CHK2) is phosphorylated by ataxia telangiectasia mutated (ATM), and activation of the CHK1 and CHK2 kinases leads to phosphorylation of downstream factors to trigger cellular responses to DNA damage, including transcriptional regulation, abnormal energy consumption, cell‐cycle arrest or delay, DNA repair, or cell death under extensive DNA damage conditions (Zeman and Cimprich, 2014). Inactivation of these checkpoint pathways is associated with tumorigenesis and malignant transformation (Bartek and Lukas, 2003). However, CHK1 itself is rarely mutated in cancer (Zhang and Hunter, 2014). It is possible that CHK1 loss‐of‐function mutations do not allow for clonal expansion of cancer cells, and therefore, these cells die off early during tumorigenesis. Furthermore, CHK1 overexpression is associated with tumor growth potentially through increased survival in response to replication stress, as well as increased resistance to chemotherapy (Zhang and Hunter, 2014), suggesting that CHK1 levels play a critical role during tumorigenesis. Therefore, it is important to elucidate mechanistically how CHK1 protein abundance is controlled, particularly under pathological tumor microenvironment conditions, which could provide a sound rationale for targeting tumors via correcting their growth environment.

Checkpoint kinase 1 kinase activity is controlled through multiple pathways including phosphorylation as well as ubiquitination. In fact, these modifications have been shown to work in concert to regulate the activation as well as subsequent inactivation through degradation of CHK1. For instance, activating phosphorylation of CHK1 at Ser^317^ and Ser^345^ in response to DNA damage and replication stress is also utilized by the E3 ubiquitin ligase machinery to recognize CHK1 as a substrate for ubiquitination and subsequent degradation, thereby turning off the checkpoint signal (Leung‐Pineda *et al*., 2009; Zhang *et al*., 2005; Zhang *et al*., 2009). In addition to regulation of CHK1 degradation in response to genotoxic stress, CHK1 is also degraded in response to glucose deprivation, which appears independent of phosphorylation at Ser^317^ and Ser^345^ (Kim *et al*., 2011). While CHK1 has been previously shown to be targeted by a variety of E3 ubiquitin ligases including CRL4 (CDT2) (Huh and Piwnica‐Worms, 2013), SCF^Fbx6^ (Zhang *et al*., 2009), and Cul4A/DDB1 (Leung‐Pineda *et al*., 2009), the E3 ubiquitin ligase responsible for regulating CHK1 under glucose deprivation remains elusive. The linkage between reduced CHK1 functionality and glucose deprivation may provide an explanation, in part, for why the tumor microenvironment, which often contains low glucose concentrations, enhances genomic instability and mutation rates during tumorigenesis (McGranahan and Swanton, 2017; Papp‐Szabo *et al*., 2005; Yuan and Glazer, 1998).

Here, we report that an AMP‐activated protein kinase (AMPK)/beta‐transducin repeat‐containing protein (β‐TrCP) signaling pathway is involved in degrading CHK1 under glucose deprivation conditions. Specifically, glucose deprivation activates AMPK, which phosphorylates CHK1 at Ser^280^ for subsequent β‐TrCP binding and ubiquitination by SCF^β‐TrCP^ for targeted degradation. Our study suggests that the AMPK‐CHK1‐β‐TrCP axis plays a role in regulating cellular levels of CHK1 under tumor growth environments that favor tumorigenesis.

## 2 Materials and methods

### 2.1 Cell culture and transfection

HEK293, MDA‐MB‐231, SK‐BR3, and H1299 cells were cultured in DMEM high‐glucose media (C11995500BT from Gibco, Carlsbad, CA, USA) containing 10% fetal bovine serum (FBS), 1% penicillin/streptomycin. For glucose deprivation, we washed the cells twice with PBS followed by culturing cells in glucose‐free DMEM (11966‐025 from Gibco) containing 10% FBS, 1% penicillin/streptomycin, and 1 mM sodium pyruvate. Cell transfections were performed using Lipofectamine 3000 (for siRNA) or PolyJet In Vitro DNA Transfection Reagent (for plasmid DNA). AMPK double‐knockout (DKO) MEFs were provided by Zong‐Ping Xia.

### 2.2 Antibodies and chemicals

Antibodies were obtained from commercial sources: β‐Actin (A5441), HA (11867423001), FLAG (F1804 and F7425 from Sigma, St. Louis, MO, USA), CHK1 (SC‐8408 from Santa Cruz Biotechnology, Santa Cruz, CA, USA), CUL1 (SC‐11384 from Santa Cruz Biotechnology), NEDD8 (ab81264 from Abcam, Cambridge, MA, USA), p‐CHK1 Ser280 (2347), CHK2 (2662), p‐AMPK Thr172 (2535), AMPK (D5A2), p‐AKT Ser473 (4060), AKT (4691), p‐ERK Thr202/Tyr204 (9101), ERK (4696), p‐GSK3β Ser9 (9323), GSK3α/β (5676), p‐ACC Ser79 (3661), ACC (3676), PARP (9542), β‐TrCP1 (4394), MEK1/2 (9216), and Vimentin (3390) (Cell Signaling Technology, Danvers, MA, USA). Compounds were obtained from commercial sources: MLN4924 (B1036 from Apexbio, Houston, TX, USA), Compound C (S7840 from Selleckchem, Houston, TX, USA), AICAR (S1802 from Selleckchem), UCN‐01 (ALX‐380‐222‐MC25 from Enzo Biochem), and LY294002 (L9908 from Sigma).

### 2.3 Constructs

The CHK1‐HA, β‐TrCP1 wild‐type, and β‐TrCP1‐ΔF plasmids were previously described (Zhao *et al*., 2011). The AMPK‐WT‐MYC‐α2 and AMPK‐DN‐MYC‐α1 plasmids were a kind gift from Wei Liu and subcloned into pIRES2‐EGFP. Mutations were generated using QuikChange Site‐Directed Mutagenesis Kit (Agilent, Santa Clara, CA, USA) according to the manufacturer's instructions. FLAG‐FBXW7, FLAG‐SKP2, and His‐Ubiquitin were described previously (Dai *et al*., 2014; Inuzuka *et al*., 2012; Li *et al*., 2016).

### 2.4 Immunoprecipitation and Immunoblotting

For experiment in Fig. 2C, HEK293 were transfected with FLAG‐tagged β‐TrCP1, FBXW7, or SKP2 in pIRES2‐EGFP and grown under glucose deprivation conditions combined with 10 μm MG132 for 12 h before harvesting. Cells were lysed in lysis buffer (50 mM Tris/HCl (pH 8.0), 150 mM NaCl, 0.5 mM EDTA, 0.5% NP‐40) supplemented with 1X Halt Protease and Phosphatase Inhibitor Cocktail (Thermo Scientific, Waltham, MA, USA) and incubated for 30 min at 4 °C. Lysates were cleared by centrifugation at 21 000 ***g*** for 10 min at 4 °C. Cleared lysates were then subjected to immunoprecipitation (IP) with bead‐conjugated FLAG antibody (Sigma) with constant rotation at 4 °C for 4 h. The immunoprecipitates were washed with lysis buffer six times for 5 min and assessed by immunoblotting (IB).

For experiment in Fig. 2D, cells were grown under normal glucose or glucose deprivation conditions together with MG132 (20 μm) for 2 h. Following lysis, IPs were carried out by adding either 10 μL mouse IgG (SC‐2025 from Santa Cruz Biotechnology) or 20 μL CHK1 Ab (SC‐8408 from Santa Cruz) to each sample and incubating with constant rotation at 4 °C for 16 h. Protein G beads (17‐0618‐01 from GE Healthcare, Chicago, IL, USA) were added to each IP and incubated for an additional 2 h at 4 °C and subsequently assessed by IB.

For direct IB analysis, cells were washed twice in PBS and lysed in RIPA buffer with phosphatase inhibitors and protease inhibitor cocktail.

### 2.5 *In vivo* ubiquitination assay

HEK293 cells were transfected with indicated plasmids and grown in glucose‐free media with 20 μm MG132 for the indicated time points. Cells were then lysed in 6 M guanidine denaturing solution and incubated with Ni‐NTA resin (Qiagen, Valencia, CA, USA) as described previously (Zhao *et al*., 2011).

### 2.6 *In vitro* kinase assay

*In vitro* kinase assays were performed as previously described (Su *et al*., 2017) with minor modifications. Immunoprecipitated HA‐CHK1 was eluted from HA‐agarose beads by incubating with 80 μL (1 mg·mL^−1^) HA peptide while shaking at 1100 rpm for 45 min at 4 °C. HEK293 cells were transfected with FLAG vector or FLAG‐AMPK WT/DN and cultured in glucose‐free media for 12 h before harvest. Immunoprecipitated FLAG vector or FLAG‐AMPK WT/DN was incubated with HA‐CHK1 (1 μL) in kinase reaction buffer (50 mM HEPES, 80 mM NaCl, 1 mM dithiothreitol, 0.02‰ Brij‐35, 0.1 mM ATP, 0.3 mM AMP, and 25 mM MgCl_2_) at 30 °C while shaking at 1100 rpm for 90 min. Reactions were stopped by addition of 15 μL loading buffer and boiled at 100 °C for 8 min. For reactions involving Compound C treatment, HEK293 cells were transfected with control vector or FLAG‐AMPK WT for 48 h and pretreated with or without 20 μm Compound C for 1 h. Cells cultured in glucose‐free media were pretreated with or without 20 μm Compound C for 4 h before harvest. Cells were processed and reactions were carried out as described above.

### 2.7 RNA isolation and RT‐PCR

RNA was isolated from cells by TRIzol followed by analysis for CHK1 mRNA levels using SYBR Green RT‐PCR Kit (Thermo Fisher). The primers used for qPCR are as follows: GAPDH: For AGGGCATCCTGGGCTACAC and Rev GCCAAATTCGTTGTCATACCAG; and CHK1: For CTGCAATGCTCGCTGGAGAAT and Rev GGGCTGGTATCCCATAAGGAAAGA. Relative expression levels were determined by normalization to the housekeeping gene *GAPDH* using the comparative Ct (2^ΔΔCt^) method.

### 2.8 siRNA and shRNA silencing

Cells were transfected with the following siRNA oligonucleotides by Lipofectamine 3000: siCtrl: ATTGTATGCGATCGCAGAC; siβ‐TrCP (recognizes both β*‐TrCP1* and β*‐TrCP2*): AAGTGGAATTTGTGGAACATC; siLKB1: CATCTACACTCAGGACTTCAC; siAKT1: TGCCCTTCTACAACCAGGA; and siCUL1: GGTCGCTTCATAAACAACA.

To knock down AMPK, we utilized two separate shRNAs, one targeting AMPK α1 and the other targeting AMPK α2. Each shRNA was individually packaged into lentivirus, and cells were subsequently infected with a mixture of each lentiviral preparation. Sequences of the AMPK α1 and AMPK α2 shRNAs are as follows: PRKAA1: CAAAGTCGACCAAATGATA; and PRKAA2: GCATACCATCTTCGTGTAAGA.

All shRNA vectors were packaged into lentivirus that were subsequently incubated with target cells followed by selection with puromycin (Invitrogen) to remove uninfected cells. Viral production, cellular transduction, and selection were performed as previously described (Li *et al*., 2016).

### 2.9 Detection of CHK1 phosphorylation sites *in vivo*

To map CHK1 phosphorylation sites *in vivo*, nanoscale microcapillary reversed‐phase liquid chromatography–tandem mass spectrometry (LC‐MS/MS). HEK293T cells were transfected with HA‐CHK1 without or with FLAG‐AMPK. Forty‐eight hours posttransfection, cells were transferred to glucose‐free media and 5 μm MG132 for 16 h to trigger AMPK activation but block the CHK1 degradation. Cells were then harvested and lysed, and HA‐CHK1 was immunoprecipitated with agarose‐conjugated HA antibody (Sigma). HA‐CHK1 was then subjected to LC‐MS/MS as previously described (Gao *et al*., 2011).

### 2.10 Subcellular fractionation

MDA‐MB231, H1299, and HEK293T cells are cultured in normal glucose or in glucose‐free media for 3 h prior to harvest. Cells were fractionated into nuclear and cytoplasm fractions using the Cell Fractionation Kit (9038 from Cell Signaling Technology). Proteins were separated and immunoblotted as described above.

### 2.11 Statistical analysis

Paired two‐tailed Student's t‐test was used for statistical analysis for data presented in Fig. 4B.

## 3 Results

### 3.1 CHK1 protein abundance is reduced in response to glucose deprivation

**Figure 1:**
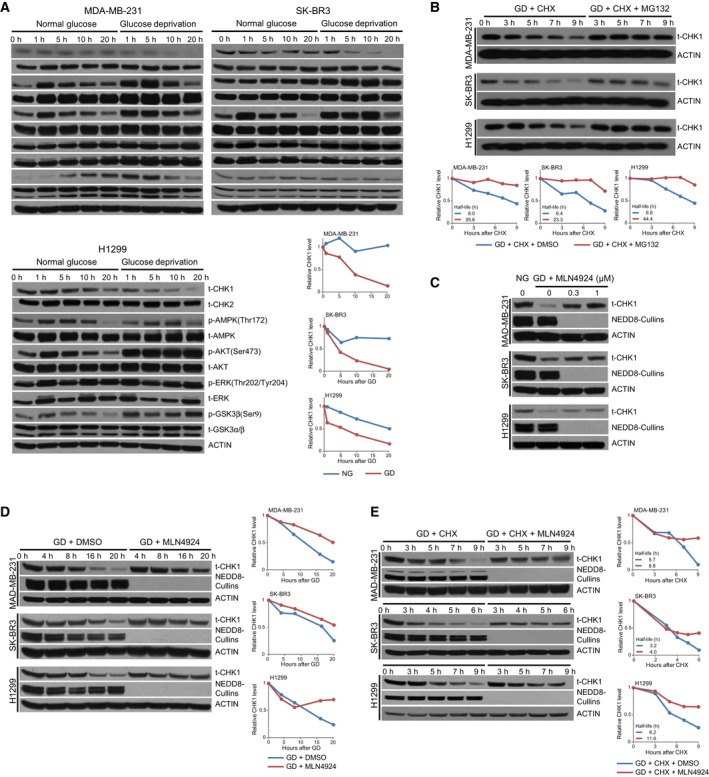
Cullin‐RING E3 ligases control CHK1 stability in response to glucose deprivation. (A) MDA‐MB‐231, SK‐BR3, and H1299 cells were grown in normal glucose and glucose‐free media and harvested at the indicated time points. Following lysis, protein was separated on SDS/PAGE and immunoblotted for CHK1, CHK2, p‐AMPK, t‐AMPK, p‐AKT, t‐AKT, p‐ERK, t‐ERK, p‐GSK3β, t‐GSK3α/β, and ACTIN. (B**)** MDA‐MB‐231, SK‐BR3, and H1299 cells were transferred to glucose‐free media containing 100 mg·mL^−1^ cycloheximide (CHX) and either DMSO or 10 μm MG132 and harvested at the indicated time points. Following lysis, protein was separated on SDS/PAGE and immunoblotted for CHK1 and ACTIN. **(**C and D**)** MDA‐MB‐231, SK‐BR3, and H1299 cells were grown in normal glucose and glucose‐free media with increasing concentrations of MLN4924 for 12 h (C), or with either DMSO or 1 μm MLN4924 for the indicated time points (D). Following lysis, protein was separated on SDS/PAGE and immunoblotted for CHK1, NEDD8, and ACTIN. (E**)** MDA‐MB‐231, SK‐BR3, and H1299 cells were grown in glucose‐free media with 100 mg·mL^−1^ cycloheximide (CHX) and either DMSO or 1 μm MLN4924 and harvested at the indicated time points. Following lysis, protein was separated on SDS/PAGE and immunoblotted for CHK1, NEDD8, and ACTIN. ‘t‐’ denotes antibodies that recognize total protein rather than a specifically modified version.

Previous studies have shown that CHK1 is targeted for degradation following DNA damage as well as under glucose deprivation (Kim *et al*., 2011), but detailed molecular mechanisms behind this degradation remain elusive. We first assessed the regulation of CHK1 protein abundance by glucose deprivation in breast cancer cell lines MDA‐MB‐231 and SK‐BR3 and in the lung cancer cell line H1299 by growing cells in glucose‐free media over a period of 20 h. We found that glucose deprivation reduced levels of CHK1 protein in each cell line starting as early as 1 h of glucose deprivation in MDA‐MB‐231 and H1299 cells and was more pronounced after 10–20 h of glucose deprivation in all cells tested without affecting the levels of CHK2, AMPK, AKT, ERK, or GSK3α/β (Fig. 1A). To assess whether degradation of CHK1 was through a proteasome‐dependent pathway, each of the cell lines was placed under glucose deprivation and treated with cycloheximide (CHX), which blocks new protein synthesis, in the absence or presence of the proteasome inhibitor MG132, and harvested at the indicated time points. We observed that the loss of CHK1 in response to glucose deprivation was blocked by MG132 (Fig. 1B). These results are consistent with recent results demonstrating that glucose deprivation leads to degradation of CHK1 (Kim *et al*., 2011) in each of the cell lines tested.

Glucose deprivation or inhibition of glycolysis has been shown to cause increased degradation of HuR, Sp1, and cyclin D1 through the E3 ubiquitin ligase beta‐transducin repeat‐containing protein (β‐TrCP) (Chu *et al*., 2012; Wei *et al*., 2008; Wei *et al*., 2009). β‐TrCP is a member of the Cullin‐RING E3 ubiquitin ligases (CRL), which require neddylation of the Cullin scaffold protein for their activity. Therefore, we treated cells with the neddylation inhibitor MLN4924 to determine whether glucose deprivation was controlling CHK1 degradation through a CRL‐based mechanism. We observed that MLN4924 treatment blocked the loss of CHK1 protein caused by glucose deprivation in the three cell lines tested in a dose (Fig. 1C)‐ and time‐dependent manner (Fig. 1D). By assessing the rate of CHK1 protein decline following CHX treatment, we found that MLN4924 blocked the effect of glucose deprivation on CHK1 protein abundance by prolonging the protein half‐life of CHK1 (Fig. 1E). These results indicate that a Cullin‐RING E3 ubiquitin ligase regulates CHK1 stability in response to glucose deprivation.

### 3.2 β‐TrCP1 interacts with CHK1 to regulate its degradation in response to glucose deprivation

**Figure 2:**
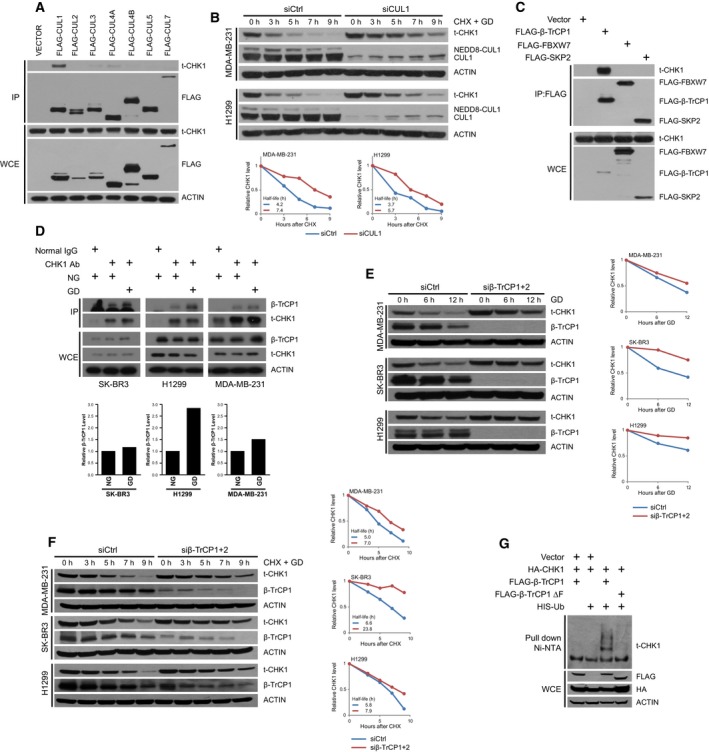
SCF ^β‐Tr^ ^CP^ targets CHK1 for degradation under glucose deprivation. (A) HEK293 cells were transfected with vectors encoding indicated FLAG‐tagged Cullin proteins. Cells were lysed and immunoprecipitated with agarose‐conjugated FLAG antibody. Immunoprecipitates and whole‐cell extracts were separated on SDS/PAGE and immunoblotted for CHK1, FLAG, and ACTIN. (B) MDA‐MB‐231 and H1299 cells transfected with siCtrl or siCUL1 and grown in glucose‐free media with 100 mg·mL^−1^ cycloheximide (CHX) for the indicated time points. Cells were harvested and lysed and protein separated on SDS/PAGE and immunoblotted for CHK1, NEDD8, and ACTIN. (C) HEK293 cells transfected with empty vector, FLAG‐β‐TrCP1, FLAG‐FBXW7, or FLAG‐SKP2 and grown in glucose‐free media and treated with 10 μm MG132 for 12 h. Cells were lysed and immunoprecipitated with agarose‐conjugated FLAG antibody. Immunoprecipitates and whole‐cell extracts were separated on SDS/PAGE and immunoblotted for CHK1, FLAG, and ACTIN. (D) MDA‐MB‐231, SK‐BR3, and H1299 were treated with normal glucose or glucose‐free media along with 20 μm MG132 for 2 h before harvest. Cells were lysed and immunoprecipitated with either IgG or CHK1 antibody. Immunoprecipitates and whole‐cell extracts were separated on SDS/PAGE and immunoblotted for β‐TrCP1, CHK1, and ACTIN. (E) MDA‐MB‐231, SK‐BR3, and H1299 cells transfected with siCtrl or siβ‐TrCP1 + 2 and grown in glucose‐free media for the indicated time points. Cells were harvested and lysed and protein separated on SDS/PAGE and immunoblotted for CHK1, β‐TrCP1, and ACTIN. (F) MDA‐MB‐231, SK‐BR3, and H1299 cells transfected with siCtrl or siβ‐TrCP1 + 2 and grown in glucose‐free media with 100 mg·mL^−1^ CHX for the indicated time points. Cells were harvested and lysed and protein separated on SDS/PAGE and immunoblotted for CHK1, β‐TrCP1, and ACTIN. (G) HEK293 cells were transfected with HA‐CHK1, FLAG‐β‐TrCP1 WT, FLAG‐β‐TrCP1 ΔF, and His‐Ub as indicated. Cells grown in glucose‐free media and treated with 20 μm MG132 for 5.5 h were lysed under denaturing conditions, and Ub‐conjugated proteins were pulled down with Ni‐NTA resin. Pull‐downs and whole‐cell extracts were separated on SDS/PAGE and immunoblotted for CHK1, FLAG, HA, and ACTIN.

Our results suggest that glucose deprivation regulates CHK1 protein abundance through increasing its degradation via activating a CRL pathway. To determine which Cullin is involved in regulating CHK1 degradation, we carried out co‐immunoprecipitation assays to determine which Cullin scaffold protein interacted with CHK1. We assessed interaction between CHK1 and each of the seven Cullin factors and found that CUL1 specifically interacted with CHK1 in HEK293 cells (Fig. 2A). Furthermore, depleting CUL1 reduced the degradation of CHK1 in response to glucose deprivation (Fig. 2B). Cullin 1 is a scaffold protein for Skp1‐Cullin‐F‐box (SCF) E3 ubiquitin ligase complexes, which target proteins for ubiquitination by recognizing and binding to degron domains in substrate molecules through the substrate recognition subunit (the F‐box protein) within the SCF complexes (Lau *et al*., 2012). Of the ~70 F‐box proteins that function in SCF complexes, β‐TrCP, FBXW7 (F‐box/WD repeat‐containing protein 7), and SKP2 (S‐phase kinase‐associated protein 2) are the most extensively studied, and each has strong links by their regulating numerous oncogenes and tumor suppressors for degradation (Wang *et al*., 2014). Given that β‐TrCP, a substrate recognition protein for the SCF complex, has previously been shown to regulate stability of various proteins in response to glucose deprivation (Chu *et al*., 2012; Wei *et al*., 2008, 2009) and that β‐TrCP forms a SCF complex with Cullin 1 (Zhao and Sun, 2013), we went on to test whether β‐TrCP also interacted with CHK1. We observed that β‐TrCP interacted with CHK1, whereas other key F‐box proteins that serve as a substrate recognition protein in SCF complexes, namely FBXW7 and SKP2, do not interact with CHK1 (Fig. 2C). More importantly, we observed that CHK1 interacted with β‐TrCP at the endogenous level, which was enhanced by culturing the cells under glucose deprivation conditions (Fig. 2D). Furthermore, glucose deprivation also promoted ubiquitination of CHK1 (Fig. S1), suggesting glucose deprivation resulted in enhanced binding and subsequent ubiquitination of CHK1, promoting its degradation.

Moreover, depletion of β*‐TrCP1* and β*‐TrCP2* using a siRNA that targets both mRNAs also blocked the loss of CHK1 protein in response to glucose deprivation (Fig. 2E), which was due in part to prolonging the half‐life of CHK1 (Fig. 2F). We also observed that co‐expressing CHK1 with β‐TrCP1 resulted in an increase in ubiquitinated species of CHK1, which was not observed when we expressed a form of β‐TrCP1 lacking the F‐box domain (β‐TrCP1‐ΔF) (Fig. 2G). β‐TrCP1‐ΔF is a dominant‐negative mutant that is unable to incorporate into the SCF E3 ligase but maintains the capacity to bind to its substrates (Yamoah *et al*., 2008). These results combined indicate that CHK1 interacts with, and is ubiquitinated by, β‐TrCP in response to glucose deprivation, suggesting that β‐TrCP1 is a major regulatory mechanism controlling CHK1 degradation due to glucose starvation.

### 3.3 Degradation of CHK1 is largely dependent on a β‐TrCP degron domain

**Figure 3:**
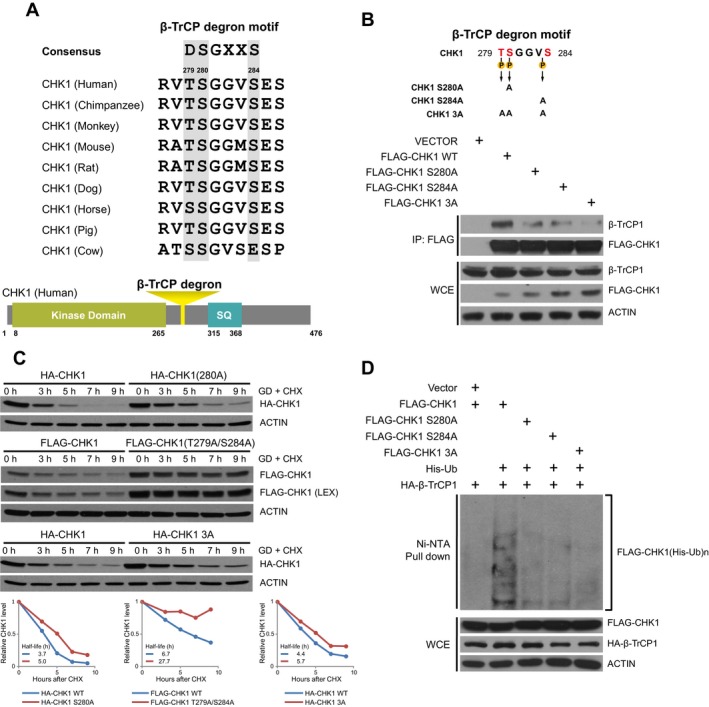
Degradation of CHK1 is largely dependent on a β‐TrCP1 degron domain. (A) CHK1 has a putative β‐TrCP1 degron motif at amino acid 279‐TSGGVS‐284 in human, which is conserved among vertebrates. (B) Diagram of mutants of β‐TrCP1 degron motif in CHK1 tested in panels B–D (top). HEK293 cells transfected with empty vector, FLAG‐CHK1 WT, FLAG‐CHK1 S280A, FLAG‐CHK1 S284A, or FLAG‐CHK1 T279A/S280A/S284A (3A). Cells were then transferred to glucose‐free media and treated with 20 μm MG132 for 6 h. Cells were lysed and immunoprecipitated with agarose‐conjugated FLAG antibody. Immunoprecipitates and whole‐cell extracts were separated on SDS/PAGE and immunoblotted for β‐TrCP1, FLAG, and ACTIN (bottom). (C) HEK293 cells were transfected with FLAG‐CHK1 WT or FLAG‐CHK1 T279A/S284A, HA‐CHK1 or HA‐CHK1 T279A/S280A/S284A, or HA‐CHK1 S280A and grown in glucose‐free media with 100 mg·mL^−1^ CHX for the indicated time points. Cells were harvested and lysed and protein separated on SDS/PAGE and immunoblotted for FLAG, HA, and ACTIN. (D) HEK293 cells were transfected with HA‐β‐TrCP1 with FLAG‐CHK1, FLAG‐CHK1 S280A, FLAG‐CHK1 S284A, or FLAG‐CHK1 T279A/S280A/S284A, and His‐Ub as indicated. Cells were grown in glucose‐free media along with 20 μm MG132 for 6 h and were lysed under denaturing conditions. Ub‐conjugated proteins were pulled down with Ni‐NTA resin. Pull‐downs and whole‐cell extracts were separated on SDS/PAGE and immunoblotted for HA, FLAG, and ACTIN.

Skp1‐Cullin‐F‐box complexes typically recognize substrates for ubiquitination through binding to a degron motif in the substrate protein recognized by the substrate recognition protein; for instance, β‐TrCP typically recognizes substrate which contains a DSGxx(x)S degron (Lau *et al*., 2012). By scanning the CHK1 protein sequence, we found that CHK1 contained a highly conserved amino acid sequence conforming to the β‐TrCP degron motif consensus, (**T/S)SGxxS (**located at amino acids 279–284 in human CHK1) (Fig. 3A), which is similar to the classical degron recognized by β‐TrCP. Furthermore, it has been reported that β‐TrCP could bind a TSGxxS motif (Setoyama *et al*., 2007). To assess the contribution of the putative β‐TrCP degron domain in CHK1 in regulating its binding to, and degradation by, β‐TrCP in response to glucose deprivation, we mutated key amino acids within the degron domain, namely Ser^280^ to alanine (S280A), Ser^284^ to alanine (S284A), Thr^279^ and Ser^284^ to alanine (T279A/S284A), or a triple mutant (T279A/S280A/S284A), and assessed interaction with β‐TrCP and stability of these CHK1 mutants. We found that mutation of these conserved amino acids within the β‐TrCP degron motif reduced the interaction of CHK1 with β‐TrCP1 under glucose deprivation conditions (Fig. 3B). On the contrary, mutating these same residues in CHK1 to glutamic acid (S to E) to mimic phosphorylation led to both an increase in interaction with β‐TrCP and an increase in ubiquitination (Fig. S2A,B). Furthermore, when we subjected cells to glucose deprivation in the presence of CHX, we observed that the mutant CHK1 had a prolonged half‐life in response to glucose deprivation (Figs 3C and S2C). Finally, mutation of the β‐TrCP degron domain in CHK1 resulted in a decrease in CHK1 ubiquitination in cells (Fig. 3D). These results together indicate that β‐TrCP regulates CHK1 degradation in response to glucose deprivation by binding to a β‐TrCP degron domain within CHK1.

### 3.4 AMPK regulates glucose deprivation‐induced degradation of CHK1

**Figure 4:**
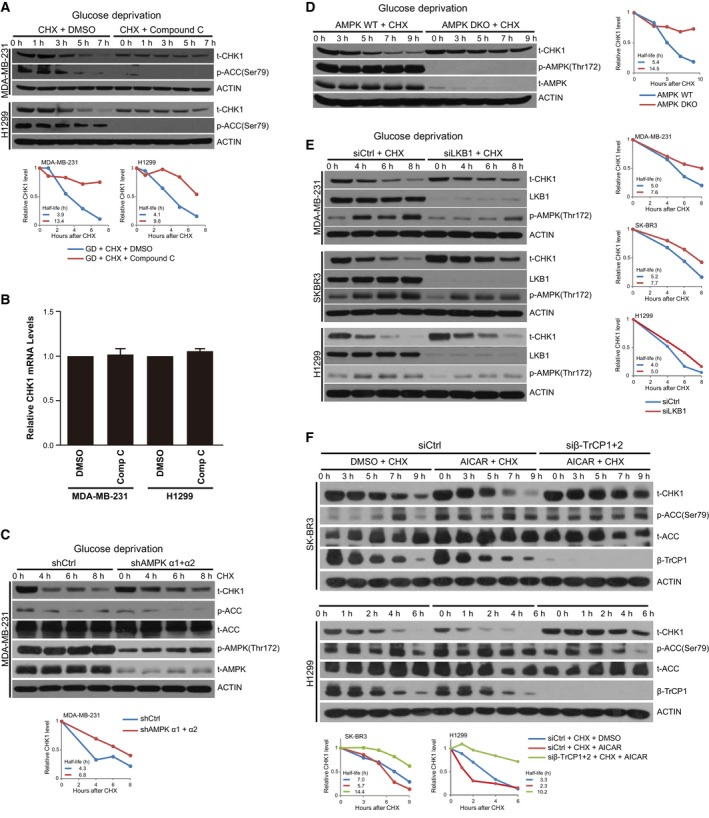
Inhibition of AMPK blocks glucose deprivation‐induced CHK1 degradation. (A) Indicated cells were grown in glucose‐containing media and treated with or without Compound C at 20 μm (MDA‐MB‐231 cells) or 40 μm (H1299 cells) for 1 h, and then transferred into glucose‐free media with Compound C at the dose indicated above for 3.5 h, upon which time CHX was added to 100 mg·mL^−1^ for the indicated time points. Cells were harvested and lysed, and protein was separated on SDS/PAGE and immunoblotted for CHK1, p‐ACC, and ACTIN. (B) Cells treated as in (A) were harvested prior to CHX treatment, and RNA was extracted and subjected to RT‐PCR for *Chek1*. Data generated from three independent replicates; error bars represent SEM, and a paired two‐tailed Student's *t*‐test was used to assess statistical significance. (C) MDA‐MB‐231 cells were transfected with shCtrl or a mixture of shRNAs targeting AMPK α1 and AMPK α2. Cells were transferred to glucose‐free media and harvested at the indicated time points following addition of 100 mg·mL^−1^ CHX. Cells were harvested and lysed and protein separated on SDS/PAGE and immunoblotted for CHK1, p‐ACC, t‐ACC, p‐AMPK, t‐AMPK, and ACTIN. (D) WT and double AMPK KO (DKO) MEFs were grown in glucose‐free media for the indicated time points with 100 mg·mL^−1^ CHX. Cells were harvested and lysed and protein separated on SDS/PAGE and immunoblotted for CHK1, p‐AMPK, t‐AMPK, and ACTIN. (E) MDA‐MB‐231, SK‐BR3, and H1299 cells were transfected with siCtrl or siLKB1 and grown in glucose‐free media for the indicated time points with 100 mg·mL^−1^ CHX. Cells were harvested and lysed and protein separated on SDS/PAGE and immunoblotted for CHK1, LKB1, p‐AMPK, and ACTIN. (F) SK‐BR3 and H1299 cells expressing siCtrl or siβ‐TrCP1 + 2 were treated with DMSO or 0.5 mm AICAR for 12 h, and subsequently, CHX was added to 100 mg·mL^−1^ for the indicated time points. Cells were harvested and lysed, and protein was separated on SDS/PAGE and immunoblotted for CHK1, p‐ACC, t‐ACC, β‐TrCP1, and ACTIN.

AMP‐activated protein kinase is a key energy sensor and is activated under a variety of conditions including glucose starvation (Laderoute *et al*., 2006), and is composed of a catalytic α‐subunit and two regulatory subunits (β and γ) (Hardie *et al*., 2012). Upon reduction in cellular energy levels, AMPK promotes ATP production by increasing the expression and activity of factors involved in catabolism while conserving energy by reducing biosynthetic pathways (Hardie *et al*., 2012). To determine whether AMPK activity is important for regulating glucose deprivation‐induced degradation of CHK1, we treated cells undergoing glucose deprivation with the AMPK inhibitor Compound C (Hawley *et al*., 2002). Inhibiting AMPK by Compound C, which led to the expected reduction in ACC phosphorylation, greatly reduced the loss of CHK1 in response to glucose deprivation in MDA‐MB‐231 and H1299 cells (Fig. 4A). We did not observe a change in CHK1 protein levels in response to glucose deprivation when we inhibited PI3K/AKT pathway with LY294002 (Fig. S3A,B). Regulation of CHK1 by Compound C was not due to a change in CHK1 mRNA (Fig. 4B), suggesting that pretreating with Compound C reduced CHK1 through a posttranscriptional mechanism.

To assess whether the effect of Compound C treatment on CHK1 stability was through its regulation of AMPK, we next assessed CHK1 stability in cells where *AMPK* is depleted or knocked out. To deplete AMPK, we used two shRNAs targeting AMPK α1 and AMPK α2, respectively. shRNAs targeting AMPK α1 and AMPK α2 were each packaged into lentivirus, and cells were infected with both lentiviral preparations together to achieve knockdown of both AMPK α1 and AMPK α2. MDA‐MB‐231 cells were depleted of *AMPK* and assessed for CHK1 levels following glucose deprivation. We observed that depletion of *AMPK* blocked, in part, the reduction in CHK1 triggered by glucose deprivation (Fig. 4C), whereas we did not observe any effect on CHK1 protein in response to glucose deprivation when we depleted *AKT1* (Fig. S3C). A similar stabilization of CHK1 in response to glucose deprivation was observed in MEFs that have both *AMPK1* and *AMPK2* knocked out (Fig. 4D). Similarly, when we depleted *LKB1*, and upstream activator of AMPK, we observed reduced degradation of CHK1 in response to glucose deprivation in MDA‐MB‐231, SK‐BR3, and H1299 cells (Fig. 4E), consistent with the notion that the AMPK pathway is involved in regulating CHK1 degradation in response to glucose deprivation.

We next set out to determine whether the small‐molecule activator of AMPK, AICAR, would increase the degradation of CHK1. By pretreating SK‐BR3 and H1299 cells with AICAR, and subsequently treating with cycloheximide under normal glucose conditions, we observed that activation of AMPK increased the rate of CHK1 degradation in cells (Fig. 4F). Furthermore, by depleting β*‐TrCP1* and β*‐TrCP2* in SK‐BR3 and H1299 cells, we found that the ability of AICAR treatment to promote the degradation of CHK1 was dependent on the presence of β‐TrCP1 and/or β‐TrCP2 (Fig. 4F). Our results therefore indicate that glucose deprivation regulates CHK1 degradation through increasing the activity of AMPK, suggesting that AMPK may regulate the ability of β‐TrCP to target CHK1 for degradation, but the underlying mechanism remains elusive.

### 3.5 AMPK phosphorylates CHK1 in the β‐TrCP degron domain

**Figure 5:**
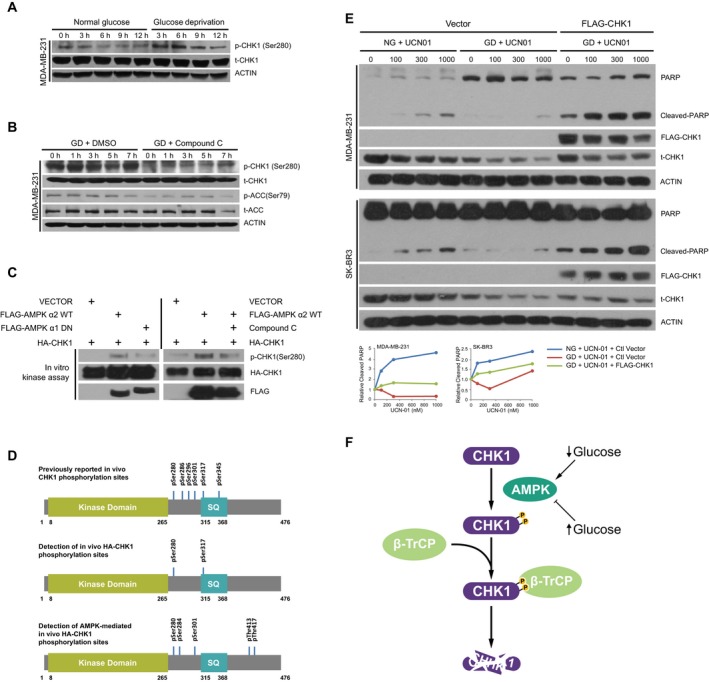
Checkpoint kinase 1 is phosphorylated by AMPK upon glucose deprivation to govern cancer cell survival. (A) MDA‐MB‐231 cells were grown in normal glucose and glucose‐free media in the presence of 10 μm MG132 and harvested at the indicated time points. Following lysis, protein was separated on SDS/PAGE and immunoblotted for p‐CHK1 (S280), t‐CHK1, and ACTIN. (B) MDA‐MB‐231 cells were pretreated with either DMSO or Compound C (20 μm) for 1 h and then transferred into glucose‐free media with Compound C at the dose indicated above for 3.5 h, followed by addition of MG132 to a final concentration of 10 μm, and harvested at the indicated time points. Following lysis, protein was separated on SDS/PAGE and immunoblotted for p‐CHK1, t‐CHK1, p‐ACC, t‐ACC, and ACTIN. (C) HEK293 cells were transfected with empty vector, FLAG‐AMPK α2 WT, FLAG‐AMPK α1 Dominant Negative (DN), or HA‐CHK1. Cells were lysed and immunoprecipitated with agarose‐conjugated FLAG antibody and HA antibody. Immunoprecipitates were processed and incubated together in an *in vitro* kinase assay in the absence or presence of Compound C as indicated and as described in the Materials and Methods section. Proteins were separated on SDS/PAGE and immunoblotted for p‐CHK1, HA, and FLAG. (D) Schematic diagram including previously identified phosphorylation sites in CHK1 (top) (Goto *et al*., 2015) of detected phosphorylation sites of CHK1 in the absence (middle) or presence (bottom) of ectopic expression of AMPK as described in the Materials and Methods section. (E) Indicated cells ectopically expressing control vector or FLAG‐CHK1 were grown in glucose‐containing or glucose‐free media with increasing concentrations of UCN‐01 for 9 h (MDA‐MB‐231) or 6 h (SK‐BR3). Following lysis, protein was separated on SDS/PAGE and immunoblotted for PARP, CHK1, and ACTIN. (F) Diagram depicting regulation of CHK1 degradation under glucose deprivation by an AMPK/β‐TrCP pathway.

Recently, the transcription factor Gli1, which is increased in many human tumors, was shown to be regulated by an AMPK/β‐TrCP pathway (Zhang *et al*., 2017b). AMPK was shown to regulate the phosphorylation of Gli1, enhancing the binding between Gli1 and β‐TrCP and the subsequent ubiquitination of Gli1. In addition, by analyzing the protein sequence of CHK1, Ser^280^ lies within an AMPK consensus phosphorylation site (**RxxS/T**) (Gwinn *et al*., 2008; Zhang *et al*., 2016). To determine whether CHK1 phosphorylation is increased in response to glucose deprivation, we grew MDA‐MB‐231 and SK‐BR3 cells in either glucose‐containing or glucose‐free conditions in which the degradation of CHK1 was blocked by treatment with MG132. Under these conditions, we observed an increase in phosphorylation of CHK1 on Ser^280^ in both cell lines following glucose deprivation (Figs 5A and S4A). We next performed this assay in the presence of the inhibitor of AMPK, Compound C, and found that inhibiting AMPK blocked the glucose deprivation‐induced increase in CHK1 phosphorylation (Fig. 5B). To determine whether AMPK purified from cells can directly phosphorylate CHK1, we pulled down AMPK and incubated it with CHK1 in an *in vitro* kinase assay. We found that AMPK pulled down from cells was able to phosphorylate CHK1, which was dependent on AMPK activity as phosphorylation was reduced by expressing a dominant‐negative version of AMPK, and also inhibited by pretreatment with the AMPK inhibitor Compound C (Fig. 5C). Furthermore, using mass spectrometry to identify residues on CHK1 that are phosphorylated by AMPK, we identified that phosphorylation on Ser284 was induced following co‐expression with AMPK (Fig. 5D). In addition, AMPK is primarily located in the cytoplasm and CHK1 is thought to be resided mainly in the nucleus, while β‐TrCP1 and β‐TrCP2 are located at nucleus and cytoplasm, respectively (Lassot *et al*., 2001). In order to assess whether all of these three proteins involved in regulating CHK1 degradation in response to glucose deprivation are localized at the same subcellular compartment, we isolated nuclear and cytoplasmic fractions, and observed that AMPK did reside mainly in the cytoplasmic fractions under normal glucose and glucose‐free conditions. Interestingly, CHK1 was present in both cytoplasmic and nuclear fractions regardless of glucose conditions (Fig. S4B). Given that Ser^280^ and Ser^284^ are within the β‐TrCP degron domain of CHK1, these results suggest that AMPK‐mediated phosphorylation may directly promote the degradation of CHK1 by β‐TrCP within the cytoplasm.

### 3.6 Degradation of CHK1 is associated with reduced cell death in response to staurosporine

Checkpoint kinase 1 is a critical regulator of cell death, and therefore, we assessed the CHK1 stability and cell death in response to treatment of MDA‐MB‐231 and SK‐BR3 cells with the kinase inhibitor UCN‐01, a staurosporine analog. We found that treating cells with increasing concentrations of UCN‐01 led to an increase in CHK1 degradation and which was further exacerbated in cells grown under glucose deprivation, resulting in enhanced cellular survival as marked by reduced levels of cleaved PARP (Fig. 5E). Enhanced cell survival in response to UCN‐01 due to glucose deprivation was reversed by ectopic expression of CHK1 (Fig. 5E). Our results therefore indicate that glucose deprivation, which is observed in many solid tumors, promotes the degradation of CHK1 through an AMPK/β‐TrCP pathway to promote cell survival (Fig. 5F).

## 4 Discussion

Checkpoint kinase 1 is a critically important regulator of genomic integrity and therefore tumorigenesis. In the present study, we have identified that CHK1 protein stability is altered during glucose deprivation, where the half‐life of CHK1 is decreased in response to low glucose. These results are consistent with previous studies demonstrating increased degradation of CHK1 in response to glucose starvation (Kim *et al*., 2011); however, the molecular mechanisms controlling CHK1 in a glucose‐dependent manner have remained elusive. Here, we report that in response to glucose deprivation, AMPK phosphorylates CHK1 at Ser^280^ within the β‐TrCP degron motif. Phosphorylated CHK1 is then recognized by β‐TrCP, followed by CHK1 ubiquitination and degradation. Our results define the AMPK‐CHK1‐β‐TrCP axis in regulation of CHK1 levels under glucose deprivation conditions.

We found that CHK1 harbors a noncanonical β‐TrCP degron motif, which, when all three potential phosphorylation sites are mutated, leads to an increase in protein stability in response to glucose deprivation. Interestingly, we observed that the S280A mutation of CHK1 had little or even an opposite effect on protein stability (which was observed in particular in the triple mutant). However, this triple mutant had reduced binding with, and ubiquitination by, β‐TrCP (Fig. 3B–D). We suspect that this increase in degradation with this mutation may be due to altered subcellular localization due to phosphorylation at Ser^280^, which has been shown to trigger nuclear localization of CHK1 (Li *et al*., 2012). Therefore, it is possible that this particular mutation may lead to an increased association with other E3 ubiquitin ligases targeting CHK1 leading to its reduced stability.

Glucose‐sensing pathways have also been shown to regulate the ability of E3 ubiquitin ligases to recognize and target proteins for ubiquitination and degradation. Glucose deprivation or inhibition of glycolysis leads to decreased stability of HuR, Sp1, and cyclin D1 through ubiquitination and subsequent degradation regulated by the E3 ubiquitin ligase β‐TrCP (Chu *et al*., 2012; Wei *et al*., 2008, 2009)**.** In addition, AMPK, which is activated by low ATP levels, is also activated in response to low glucose (Lin and Hardie, 2018; Zhang *et al*., 2017a). Interestingly, AMPK activity has been shown to regulate the ability of β‐TrCP to recognize and ubiquitinate Gli1, a transcription factor that is involved in regulating glioblastoma tumorigenesis (Zhang *et al*., 2017b).

Our finding that β‐TrCP targets CHK1 during glucose deprivation adds to a growing list of β‐TrCP substrates that are targeted by this E3 ubiquitin ligase in a glucose‐dependent manner, including HuR, Sp1, and cyclin D1 (Chu *et al*., 2012; Wei *et al*., 2008, 2009). In addition, we have shown that the AMPK pathway, a critical energy sensor, targets CHK1 for phosphorylation under glucose deprivation, suggesting that an AMPK/β‐TrCP signaling axis may be a central component of cellular response to low‐glucose conditions. It would be of interest to assess whether AMPK is also involved in linking glucose levels to degradation of HuR, Sp1, and cyclin D1 by β‐TrCP. In addition, given a potential role for an AMPK/β‐TrCP pathway regulating Gli1 (Zhang *et al*., 2017b), it would be interesting as well to test whether Gli1 stability is also regulated by glucose levels, as we have observed for CHK1. The fact that we saw limited effect by modulating Akt was somewhat surprising as Ser^280^ has been previously shown to be an Akt phosphorylation site in CHK1 (Puc *et al*., 2005). However, one might speculate that given the degron requires multiple phosphorylation sites, that phosphorylation at Ser^280^ alone under Akt activation conditions might not be sufficient to drive recognition by β‐TrCP. Moreover, another might speculate that other signal transduction pathways may regulate CHK1 degradation in either a cell type‐specific or context‐dependent manner, and therefore, assessing additional kinase pathways may be of interest to elucidate more clearly how β‐TrCP may be regulated under various conditions or contexts.

## 5 Conclusions

In summary, solid tumors exhibit glucose deprivation conditions due to increased glucose consumption as well as disorganized vascularization (Bergers and Benjamin, 2003; Skinner *et al*., 1990). Given their dependence on glucose for energy production, cancer cells must find ways to survive under these conditions, and how they do so has remained largely unknown. Here, we show that CHK1, which is a critical regulator of genomic integrity due to its role in regulating DNA damage checkpoint signaling, is downregulated under glucose deprivation conditions through an AMPK/β‐TrCP degradation pathway. Our study raises the possibility that under glucose deprivation, a hallmark of solid tumors, cells are allowed to continue cycling in an unchecked state leading to greater genomic instability and increase in mutation burden to promote cancer development.

## Conflict of interest

The authors declare no conflict of interest.

## Author contributions

YM acquired and analyzed the data and drafted the manuscript. DC and XX acquired and analyzed the data. HI, WW, and YS revised the manuscript. BJN designed the study, acquired and interpreted the data, and drafted and revised the manuscript. YZ conceived and designed the study, acquired and interpreted the data, and revised the manuscript; and all the authors have read and approved the final manuscript.

## Supporting information


**Fig. S1.** Glucose deprivation induces ubiquitination of CHK1.
**Fig. S2.** Degradation of CHK1 is largely dependent on a β‐TrCP degron domain.
**Fig. S3.** Inhibition of AKT does not influence glucose deprivation‐induced CHK1 degradation.
**Fig. S4.** CHK1 is phosphorylated by AMPK upon glucose deprivation.
