# Supplementary material for: SCFβ‐TrCP ubiquitinates CHK1 in an AMPK‐dependent manner in response to glucose deprivation
Source: Mol Oncol. 2018 Dec 3;13(2):307–21. doi: 10.1002/1878-0261.12403 (PMC6360357; doi:10.1002/1878-0261.12403)
Supplement: Supplementary file 1 — Fig. S1. Glucose deprivation induces ubiquitination of CHK1. Fig. S2. Degradation of CHK1 is largely dependent on a β‐TrCP degron domain. Fig. S3. Inhibition of AKT does not influence glucose deprivation‐induced CHK1 degradation. Fig. S4. CHK1 is phosphorylated by AMPK upon glucose deprivation. [file MOL2-13-307-s001.pdf]

SUPPLEMENTAL INFORMATION

SCF$^{\beta\text{-TrCP}}$ Ubiquitinates CHK1 in an AMPK-Dependent Manner in Response to Glucose Deprivation

Ying Ma,[1,2,3,6] Danrui Cui,[1,2,6] Xiufang Xiong,[2] Hiroyuki Inuzuka,[3,4] Wenyi Wei,[3] Yi Sun,[2,5] Brian J. North[3,#] and Yongchao Zhao[1,2,#]

[1]Key Laboratory of Combined Multi-Organ Transplantation, Ministry of Public Health, First Affiliated Hospital, Zhejiang University School of Medicine, Hangzhou, 310003 China
[2]Institute of Translational Medicine, Zhejiang University School of Medicine, Hangzhou, 310029 China
[3]Department of Pathology, Beth Israel Deaconess Medical Center, Harvard Medical School, Boston, MA 02115 USA
[4]Center for Advanced Stem Cell and Regenerative Research, Tohoku University Graduate School of Dentistry, Sendai, 980-8575 Japan
[5]Division of Radiation and Cancer Biology, Department of Radiation Oncology, University of Michigan, Ann Arbor, MI 48109 USA
[6]These authors contributed equally to this work.

[#]Correspondence
yongchao@zju.edu.cn (Y.Z.)
bnorth@bidmc.harvard.edu (B.J.N.)

Supplemental Figures:

Fig S1: Glucose deprivation induces ubiquitination of CHK1.

Fig S2: Degradation of CHK1 is largely dependent on a β-TrCP degron domain.

Fig S3: Inhibition of AKT does not influence glucose deprivation-induced CHK1 degradation.

Fig S4: CHK1 is phosphorylated by AMPK upon glucose deprivation.

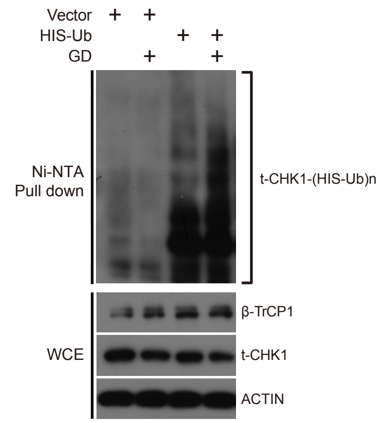

Fig. S1. Glucose deprivation induces ubiquitination of CHK1. HEK293T cells were transfected with His-Ub as indicated. Cells grown in normal glucose or glucose-free media and treated with 20 μM MG132 for 6 hrs. Cells were lysed under denaturing conditions and Ub conjugated proteins were pulled down with Ni-NTA resin. Pull downs and whole cell extracts were separated on SDS-PAGE and immunoblotted for CHK1, β-TrCP1, and ACTIN.

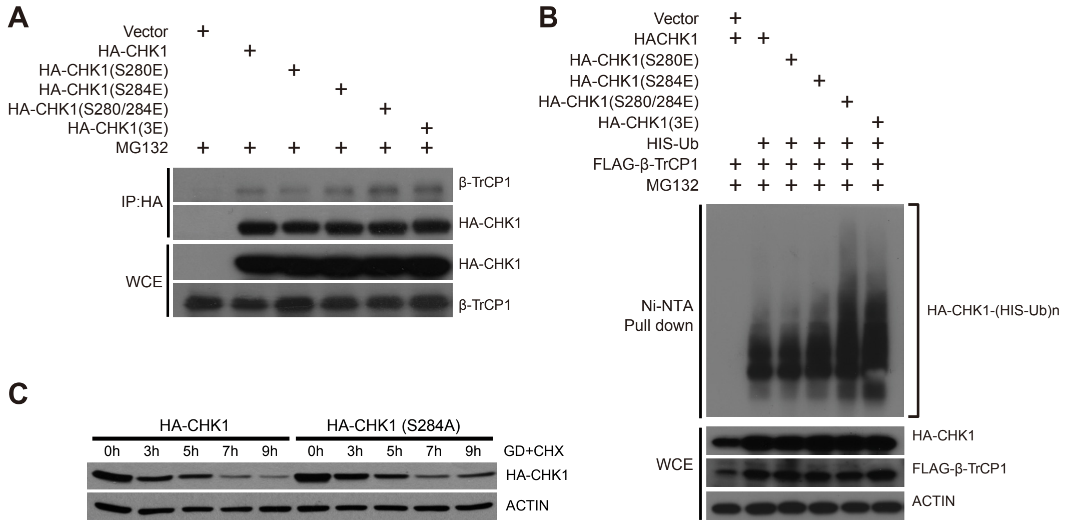

Fig. S2. Degradation of CHK1 is largely dependent on a β-TrCP degron domain. **(A)** HEK293T cells were transfected with control vector, HA-CHK1 WT, HA-CHK S280E, HA-CHK S284E, HA-CHK S280/284E, HA-CHK T279,S280/284E (3E). Cells were treated with 5 μM MG132 for 12 hrs, lysed and immunoprecipitated with agarose conjugated HA antibody. Immunoprecipitates and whole cell extracts were separated on SDS-PAGE and immunoblotted for β-TrCP1 and HA. **(B)** HEK293T cells were transfected with FLAG-β-TrCP1 along with either control vector, HA-CHK1 WT, HA-CHK S280E, HA-CHK S284E, HA-CHK S280/284E, HA-CHK T279,S280/284E (3E), and His-Ub as indicated. Cells were grown in normal glucose media and treated with 5 μM MG132 for 12 hrs before harvest. Cells were lysed under denaturing conditions and Ub conjugated proteins were pulled down with Ni-NTA resin. Pull downs and whole cell extracts were separated on SDS-PAGE and immunoblotted for HA, FLAG, and ACTIN. **(C)** HEK293 cells were transfected with HA-CHK1 WT or FLAG-CHK1 S284A and grown in glucose-free media with 100 mg/ml CHX for indicated times. Cells were harvested, lysed, and protein separated on SDS-PAGE and immunoblotted for HA and ACTIN.

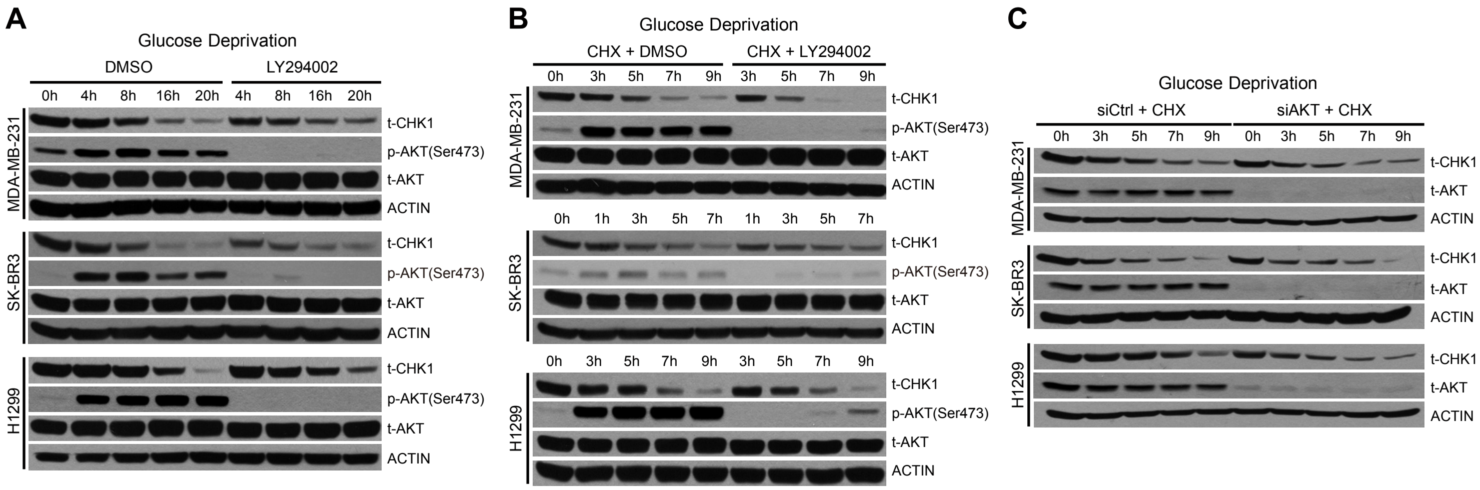

Fig. S3. Inhibition of AKT does not influence glucose deprivation-induced CHK1 degradation. (A) MDA-MB-231, SK-BR3, and H1299 cells were transferred to glucose-free media and treated with either DMSO or 25 µM LY294002 for indicated times. Cells were harvested, lysed, and protein was separated on SDS-PAGE and immunoblotted for CHK1, p-Akt, t-Akt (Ser473), and ACTIN. **(B)** MDA-MB-231, SK-BR3, and H1299 cells were grown in glucose-free media and treated with 100 mg/ml CHX and either DMSO or 25 µM LY294002 for indicated times. Cells were harvested, lysed, and protein was separated on SDS-PAGE and immunoblotted for CHK1, p-Akt (Ser473), t-Akt, and ACTIN. **(C)** MDA-MB-231, SK-BR3, and H1299 cells were transfected with siCtrl and siAKT. After 72 hrs, cells were switched to glucose-free media containing 100 mg/ml CHX for indicated times. Cells were harvested, lysed, and protein was separated on SDS-PAGE and immunoblotted for CHK1, Akt, and ACTIN.

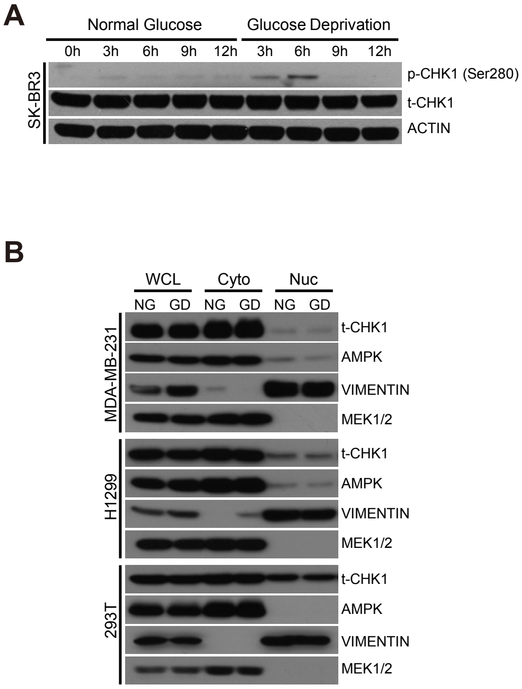

Fig. S4. CHK1 is phosphorylated by AMPK upon glucose deprivation. **(A)** SK-BR3 cells were grown in normal glucose and glucose-free media in the presence of 10 μM MG132 and harvested at indicated times. Following lysis, protein was separated on SDS-PAGE and immunoblotted for p-CHK1, t-CHK1, and ACTIN. **(B)** MDA-MB-231, H1299, and HEK293T cells were grown in normal glucose and glucose-free media for 3 hrs. Cells were then harvested and separated into various cellular compartments including whole cell (WCL), cytoplasm (Cyto), and nuclear (Nuc) fractions. Each fraction was separated on SDS-PAGE and immunoblotted for CHK1, AMPK, Vimentin (nuclear marker), and MEK1/2 (cytoplasmic marker).
